# Intra-cranial Chondroma: A Case Report and Problematic Diagnosis

**DOI:** 10.30699/IJP.2021.132377.2472

**Published:** 2020-12-26

**Authors:** Arezoo Eftekhar Javadi, Elham Nazar, Hedieh Moradi Tabriz

**Affiliations:** 1 *Department of Pathology, Sina Hospital, Tehran University of Medical Sciences, Iran*

**Keywords:** Chondroma, Intra-cranial, Cartilage, Tumor

## Abstract

**Introduction:**

Chondroma is a benign cartilaginous tumor. It is found very rarely in the head and neck.

**Case presentation:**

This report describes a 25-year-old woman who presented with generalized headache from 4 months ago. The patient underwent excisional surgery. The histological examinations revealed benign cartilage forming tumor, compatible with chondroma. The radiologic and histologic correlation confirmed the diagnosis. Based on the diagnosis, the patient received no more treatment.

**Conclusion:**

We concluded that intracranial chondroma should be included in the differential diagnosis of a calcified mass on skull imaging. Proper diagnosis is necessary for further patient management.

## Introduction

Chondroma is a benign cartilaginous tumor which can be presented in solitary or multiple forms. It originates infrequently in the head and neck (1). The cartilagineous remnants at the basisphenoid and basiocciput or the cartilagineous part of the eustachian tube seem to be the most possible origin (2). Chondroma is typically found in the middle age, and is exceptional in children (3). Clinically, the main symptom of intra-cranial chondroma is lower cranial nerve palsy; the proptosis and visual loss can also happen. More than 60% of the lesions are calcified, thus bone damage is also common. Angiography shows transposition of the vessels but no tumor stain (4). Chondromas present clinical features similar to meningiomas (5). Chondroma is a distinctive histopathological diagnosis in contrast to other cartilage-containing lesions (6). Mineralization of hyaline cartilage generally has a ring-like appearance, representative of cartilaginous tumors (7). They can be a component of Ollier’s disease or Maffucci syndrome. Sometimes, they happen as an exhibition of a generalized chondromatosis including sellar chondroma in a patient with Ollier's disease (8). Also, Maffucci syndrome is a rare disease (9) and intracranial chondromas, chondrosarcomas, chordomas, gliomas, and pituitary adenomas have been reported in link with Maffucci syndrome (10). Because the clinical presentation of such cases is non-specific and neuroimaging findings are not pathognomonic, the intracranial chondromas mimic other intracranial tumors (11). Herein, we report a chondroma originating from the dura mater in the frontoparietal region to raise the awareness about the intra-cranial chondromas.

## Case Presentation

**Figure 1:**
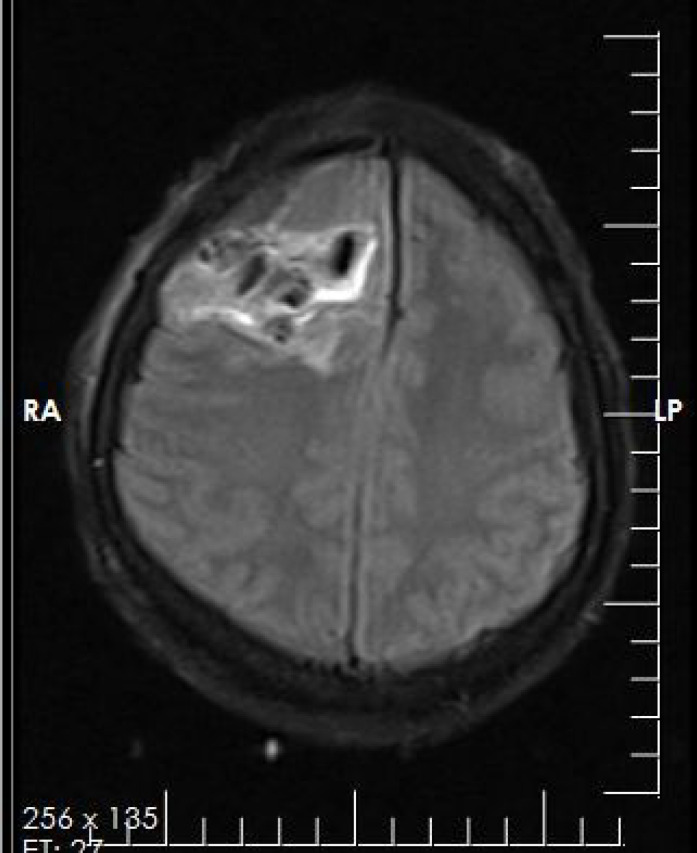
Radiologic examination shows a lobulated intra-axial mass in the right frontoparietal lobe with heterogeneous enhancement and mild peripheral edema. Shift of falx cerebri to the left side is seen

**Figure 2:**
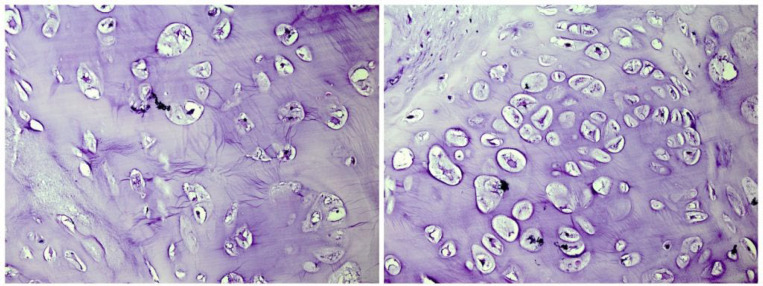
Sections show the mature cartilage with lobular architecture (x100).

A 25-year-old female referred to the Department of Neurosurgery in Sina Hospital affiliated to TUMS with a four months history of generalized headache. At first, the outpatient examination finding was unremarkable. The patient’s family history and past medical history was also unremarkable. No neurological sign was found. The patient underwent plain radiography, which showed a calcified lesion on the right frontoparietal lobe. The brain MRI (magnetic resonance imaging) with and without contrast showed a lobulated intra-axial mass in the right frontoparietal lobe with heterogeneous enhancement and mild peripheral edema. Shift of falx cerebri to the left side was seen. The radiological diagnosis was low grade glioma or meningioma (Figure 1). The diagnosis required histopathologic confirmation. The patient underwent an excisional surgery. She well tolerated the procedure without complications. The received specimen for pathology examination consisted of multiple pieces of tan firm tissue totally measuring 12×8×2.5 cm. Histological examinations revealed portions of mature cartilage with lobular architecture. No atypia or mitotic figures and necrosis were seen (Figure 2). Therefore, our case was compatible with chondroma and diagnosis was confirmed. Based on the diagnosis, the patient received no additional treatment. After 6 months of follow-up examinations, no recurrence was observed and the patient is still asymptomatic.

## Discussion

The incidence of benign bone and cartilage tumors is mainly indefinite, due to the absence of recording as well as common failure to present clinically (12). Chondroma is a distinct histopathological diagnosis in contrast to other cartilage-containing lesions and characterized by cellular atypism which was first defined by Baumuller in 1883. The zones of ossification and calcification may be recognized within the hyaline cartilage that comprises most of the lesions (13). Dahlin (1957) has identified that more than 60% of chondromas will happen in the hands and feet (14). Chondromas are uncommon intracranial tumors with an estimated incidence rate of0.2-0.3% of all intra-cranial tumors (15). The majority of intra-cranial chondromas originate from cartilage remnants in the synchondrosis at the base of the skull. Chondromas are most generally found in the sellar and parasellar areas, usually situated extra-durally (16). Several hystopathogenetic theories have been suggested such as: metaplasia of meningeal fibroblasts and perivascular meningeal tissue, traumatic or inflammatory cartilaginous activation of fibroblasts and growth of the aberrant embryonal cartilaginous remnants in the dura mater (5). Chondromas are benign cartilaginous tumors consist of mature chondrocytes related to hyaline cartilage matrix that is developed in the soft tissues, without bone or joint involvement (17). Differential diagnosis of chordoma and chondroma in the skull base is occasionally problematic. Chordomas show heterogeneous low signal intensity, and chondromas show markedly low signal intensity similar to that of CSF. The calcified or ossified portions of the chondromas were revealed as areas of moderately low intensity on images (18). The preoperative diagnosis is challenging. On magnetic resonance imaging (MRI), the chondroma seems multi-lobulated with low to medium signal intensity on T1-weighted imaging and signal hyperintensity on T2-weighted imaging (3). According to another study, these patients presented with non-specific symptoms associated with the mass effect, and imaging with non-specific characteristics which is similar to our case. thus, often meningiomas, oligodendrogliomas, and vascular malformations were in differential diagnosis (19). Although pain is often the most common presenting symptom, a 2013 study established that 75% of the patients had an alternative cause for the pain (20). Chondroma is a benign tumor of the soft tissues and malignant degeneration has not been detected and recurrence is rare (5%–18%). Thus, the treatment of choice is local excision (21). Chondroma is a benign variant, considered by the formation of mature hyaline cartilage without atypia, while chondrosarcoma is a malignant tumor that produces atypical cartilage matrix and features an infiltrative growth configuration (22). Chondrosarcoma of the skull base is an uncommon tumor with a good prognosis after surgical resection (23). But the metastasis to respiratory system secondary to chondrosarcoma is a common finding (24). Occupying the same anatomic location, the clinical presentation and radiologic features of chordoma and chondrosarcoma are relatively parallel (25). Although preoperative certain diagnosis for the skull base chondromas is difficult, approaches for diagnosis and treatment without any complication are critical (26).

## Conclusion

Chondroma is a benign cartilaginous tumor, generally reported in the extremities and is unusual in intra-cranial. When the typical clinical and radiological features are existent, diagnosis is not difficult. Problems in the differential diagnosis may occur in association with a strange setting. They present frequent uncharacteristic clinical signs that ultimately lead to insufficient treatment. Early correlation among histologic examination and imaging abnormalities could decrease the time to identify the lesion and prevent avoidable morbidity. This information is helpful for the appropriate preoperative planning.
